# Characterizing Mat Formation of Bamboo Fiber Composites: Horizontal Density Distribution

**DOI:** 10.3390/ma14051198

**Published:** 2021-03-04

**Authors:** Yu’an Hu, Mei He, Kate Semple, Meiling Chen, Hugo Pineda, Chenli Zhou, Chunping Dai

**Affiliations:** 1 Jiangxi Academy of Forestry, Nanchang 330029, China; yuan.hu@ubc.ca (Y.H.); mhe07@mail.ubc.ca (M.H.); 2 Department of Wood Science, Faculty of Forestry, University of British Columbia, 2900-2424 Main Mall, Vancouver, BC V6T 1Z4, Canada; katherine.semple@ubc.ca (K.S.); meiling.chen@ubc.ca (M.C.); ha.pineda2087@uniandes.edu.co (H.P.); zhoucl@mail.ubc.ca (C.Z.)

**Keywords:** mat formation, horizontal density distribution, density uniformity, bamboo scrimber

## Abstract

Bamboo fiber composite (BFC) is a unidirectional and continuous bamboo fiber composite manufactured by consolidation and gluing of flattened, partially separated bamboo culm strips into thick and dense panels. The composite mechanical properties are primarily influenced by panel density, its variation and uniformity. This paper characterized the horizontal density distribution (HDD) within BFC panels and its controlling factors. It revealed that HDD follows a normal distribution, with its standard deviation (SD) strongly affected by sampling specimen size, panel thickness and panel locations. SD was lowest in the thickest (40 mm) panel and largest-size (150 × 150-mm^2^) specimens. There was also a systematic variation along the length of the BFC due to the tapering effect of bamboo culm thickness. Density was higher along panel edges due to restraint from the mold edges during hot pressing. The manual BFC mat forming process is presented and found to effectively minimize the density variation compared to machine-formed wood composites. This study provides a basic understanding of and a quality control guide to the formation uniformity of BFC products.

## 1. Introduction

Bamboo is among the fastest growing woody plants in the world, capable of increasing 1 m in height per day [1]. Bamboo timber production is also rapid, on an average rotation of 4 years compared to normally 40 years for most wood species. The strength and ductility of bamboo is significantly higher than many wood species of similar density [2]. Driven by sustainability and material performance, bamboo fiber composites (BFCs) can be produced as high-strength, dimensionally stable and durable products [3,4], reducing the pressing need for tropical hardwoods and structural lumbers. Versatile BFC products are used in wind turbine blades, building panels and columns, landscape architecture, highway fences, shipping container flooring, interior/exterior flooring and decking and furniture [5,6,7,8].

**Figure 1:**
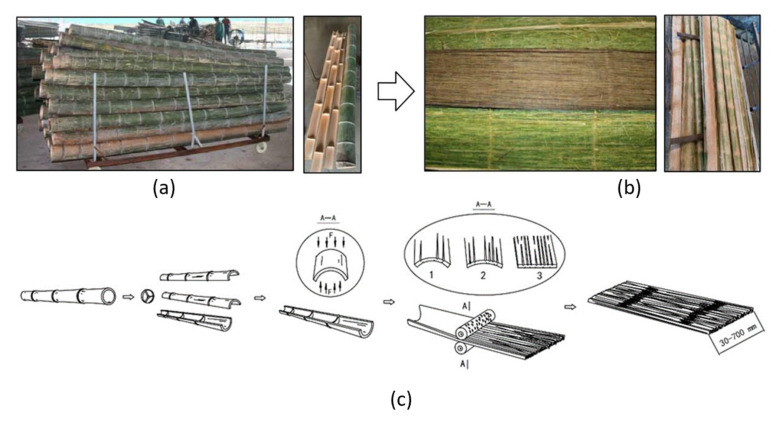
Culm conversion to “scrimmed” strips: (**a**) photos of culm splitting, (**b**) photos of scrimmed bamboo mats, and (**c**) schematic of the entire scrimming process where a bamboo culm was slipped into strips and then crushed into partially separated fiber mats through incised rollers.

The BFC described here is a novel adaptation of bamboo “scrimber” lumber, allowing large panels to be manufactured. The use of scrimmed or broomed strips in panels or laminated veneer products is a significant evolution in bamboo scrimber technology that allows mat forming to be controlled and, therefore, products to be compressed to less than the 1.25 g/cm^3^ typical of earlier bamboo scrimber billets [9,10,11,12,13]. Culms are first split into sections ranging in number from two halves to eight narrower strips which are then “scrimmed” by compressive roller crushing to flatten and break up the culm wall tissue into partially separated fiber bundles (Figure 1). The roller crushing ruptures and partially removes the outer and inner skin of the culm that is highly impervious and hampers resin bonding. The number of passes and the setting of the rollers controls the extent of fiber separation.

The scrimming process is controlled so that only small cracks are opened along the grain of the culm to allow for resin wetting and penetration, but the original integrity and strength of the bamboo culm is largely preserved. The summarized production process involves coating the scrimmed strips with liquid resin, drying and forming lengthways into a mat that is hot pressed to cure the resin and densify the product. Attention has been paid to the development and optimization of bamboo strip scrimming and brooming processes, resin application and hot pressing, e.g., [13], but the process of BFC mat formation, which is currently done manually, or panel density variation has not been investigated. The lack of an effective and practical quality control method for industrialized manufacture of bamboo bundle veneer composites currently significantly restricts their quality control and application in construction [13]. Like any composite material, BFC mats must be formed with uniform mass distribution to ensure product consistency and quality.

Wood veneer-, strand- and particle-based wood composites are formed in a fully automated process, whereas in commercial production of BFCs, the mat forming is manual. There is inevitable variation in the structure and density introduced into the mat and pressed panels arising from the macro-level non-uniformity (tapering of width and thickness) of the flattened strips and their manual arrangement. Greater density variation in the hand-formed mat necessitates greater overall compaction and densification in order to achieve acceptable bonding strength in the lower-density regions. While greater compaction leads to higher strength properties of BFC [14] and wood composite materials in general [15,16], it can also cause processing problems such as delamination due to gas pressure build-up during hot pressing [17,18,19]. Volume and thickness loss are also greater with higher mat densification, creating a trade-off between product density and culm material recovery [20]. The key to realizing optimum product performance and profit is uniform element preparation and mat formation, i.e., keeping the horizontal density variation as narrow as possible.

The concept of horizontal density distribution and its influence on properties in wood composites was first proposed by Suchsland [21,22] for particle boards. Dai and Steiner [23,24,25] developed mathematical and computer models to simulate the mat formation of wood strand-based composites [26]. Numerous studies have subsequently been carried out to experimentally evaluate horizontal density variations and the effects of wood element size, shape and orientation and to further simulate and optimize wood composite mat formation [27,28,29]. This study is the first to examine how the mat formation process and mold pressing of BFC affect horizontal density distribution. It develops a simple, practical density sampling strategy for mills to determine acceptable criteria (averages and ranges) for product quality control monitoring. A mat formation method is described whereby large elements (scrimmed, flattened bamboo strips) are arranged manually to accommodate and even out the inherent variability in the geometry and density of the raw material. Commercially produced BFC panels of three thicknesses are evaluated for density using the gravimetric method and the patterns of density variation along the edge and middle of each panel along and across the bamboo grain are analyzed. The density variation found in the BFC panels is compared with typical values found in smaller-element, machine-formed wood composites (medium density fiberboard (MDF), particle board and oriented strand board (OSB)). Key factors (target density, panel thickness, specimen location and size) affecting the horizontal density variation in hot-pressed BFC panels are presented and their implications for product quality control are discussed.

## 2. Materials and Methods

### 2.1. Materials

Bamboo: Fresh Moso bamboo culms were obtained from Yingtan city, Jiangxi province. The bamboo was 3 to 4 years old, up to 20 m in height, 80 to 100 mm in diameter with a wall thickness of 9–12 mm. Culms were cross-cut into three 2-m-long sections and split longitudinally into four sections that were roller flattened into scrimmed strips measuring 2000 × 40–100 mm^2^ width × 3–5 mm thickness. The strips were then dried to a moisture content (MC) of around 10%.

Adhesive: Phenolic resin was supplied by Beijing Taier Chemical Co., Ltd., with solids content of 46.56%, viscosity of 42 cPs and pH of 10–11.

### 2.2. Panel Fabrication and Testing

(a) Resin application and drying

Strips were dipped in a phenolic resin solution of 1 part concentrate to 8 parts water for 5 min and drained for 5 min followed by drying to 10% MC, ready for production. This is normally done over a day in summer in sunny periods and by oven at 70 °C for 3 h for winter/accelerated production. Resin dosage (solids) was approximately 13% by weight. Because the strips were scored and fractured slightly, resin was able to seep into crevices in the strips, increasing the loading compared with smooth surface coating only.

(b) Mat lay-up

**Figure 2:**
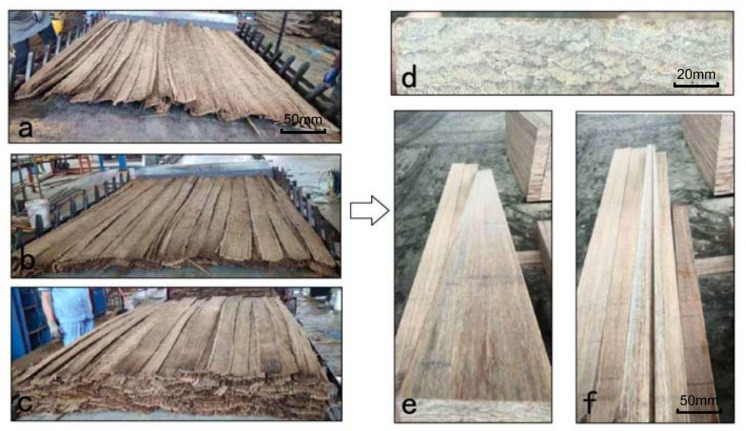
Bamboo fiber composite (BFC) formation: (**a**) first layer on caul plate, (**b**) mat build-up showing strips with minimal overlap of core strips, (**c**) finished lay-up, (**d**) cross-section of consolidated panel and (**e**,**f**) re-sawn billets from a BFC panel.

**Figure 3:**
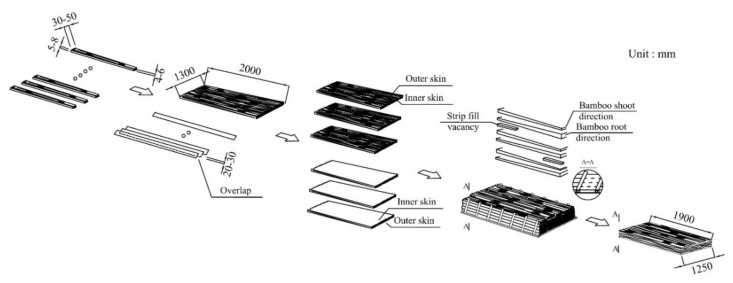
Schematic representation of elements of the mat formation process for BFC: strip dimensions, layer formation, interlayer formation, mat assembly and consolidated panel.

After drying, the resin-coated strips were arranged manually lengthwise in a large, shallow square mold, measuring 1.9 × 1.25 m^2^ and pre-compacted to reduce its height. If necessary, one or more 400-mm-long filler strips were inserted into the ends. If used at all, no more than 8–10 of these were added per panel, even for the thickest panel. The mat forming is shown in Figure 2 and Figure 3 and its optimization is described below.

Five strategies were used to reduce sources of variation during mat forming and the inherent variation in the culm materials. The manual mat lay-up process, illustrated in Figure 3, is elaborated as follows:

i. The width of the scrimmed strips was carefully controlled by the culm splitting and roller flattening processes to be as uniform as possible. Two strip widths were generally maintained: 30–50 and 60–80 mm. Here, two mat formation mechanisms may co-exist: narrow strips are good for randomization and, hence, uniformity, whereas wide strips are useful for manual placement, which can be beneficial for more uniform control of density distribution.
ii. The strips were unidirectionally oriented with no or very low angle relative to the length axis of the panel. There was partial overlap which helped to consolidate flexural strength across the panel and reduced the random arrangement of void space throughout the panel, helping to reduce horizontal density variation.
iii. The top and bottom of the panels were isometric, i.e., the bottom half of the scrimmed strips was oriented so that the outer wall of the culm faces down, and in the top half of the layers, the outer wall faces up. This is because the bamboo tissue is densest and strongest in the zone at the outer wall, which is richest in fiber bundles. The strips were also placed to overlap more in the outer, or “shell”, layers to increase flexural properties across the width of the panel.
iv. Each mat layer was counter-oriented in terms of culm length direction, i.e., the bottom (root) end of the culm was located at the opposite end of the panel in each layer. This is because the width and the culm wall thickness are greater and the average density of the culm wall is lower at the bottom end of the culm. In order to control the effect of large variation in fiber bundle thickness along the entire bamboo culm, culms were cross-cut into three 2-m-long sections (bottom, middle and top). During mat forming, only the strips from the same section were used in any one layer.
v. The mat was inspected both visually and by compression to check for gaps which are filled with shorter strips. This was to even up any low-density zones found in the ends of the mat. The loose arrangement of the strips allows for some lateral expansion of the mat during pressing, helping fill edge voids and reducing horizontal density variation.

(c) Hot pressing

Mats were hot pressed at 140 °C for around 30 min until the resin was completely cured. After pressing, the panels were kept in the hot pressing machine and cooled to 60 °C for 1/2 h before demolding. Here, cooling was needed to allow the internal gas pressure to drop before opening to avoid delamination [30,31]. After pressing, the BFC panels were then conditioned for 1~2 days prior to cutting for density measurement. The panel manufacturing process is shown in Figure 2 and Figure 3.

(d) Panel density sampling

**Figure 4:**
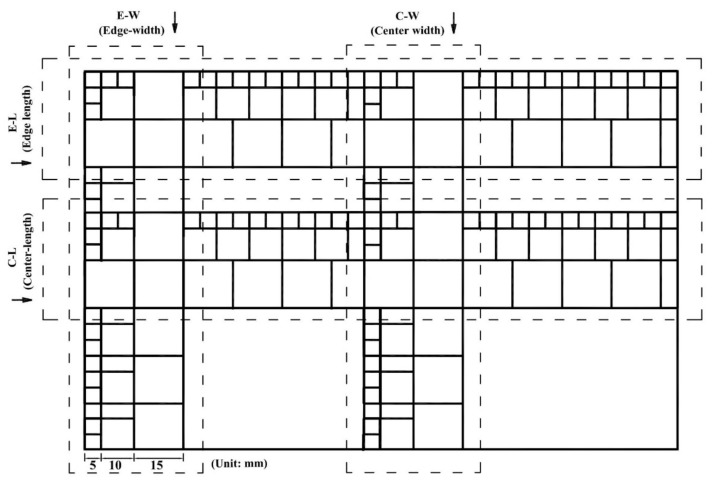
Distribution of density specimens cut from each panel.

Three different thicknesses of panel (15, 20 and 40 mm) were produced and tested. The number of layers of strips required was 6 for the 15-mm panel, 8 for the 20-mm panel and 10 to 12 for the 40-mm panel. From the finished panel, three sizes of square specimens were cut and measured for density, i.e., 50 × 50, 100 × 100 and 150 × 150-mm^2^. The cutting pattern for density specimens from each panel is shown schematically in Figure 4. Four sampling transects were used as shown on the diagram, i.e., edge-length (E-L), center-length (C-L), edge-width (E-W) and center-width (C-W). A total of 288 50 × 50-mm^2^, 114 100 × 100-mm^2^ and 108 150 × 150-mm^2^ specimens were cut from each panel and gravimetrically tested for density (air-dried basis).

The nested structure of the experiment is summarized in Table 1. A total of 510 density specimens were cut and tested for the experiment. Each specimen was measured for its length, width and thickness using Vernier calipers and its air-dried mass was recorded using an electronic balance. Density was expressed in g/cm^3^ and the standard deviation, SD, and coefficient of variation (COV, %) were calculated for each group.

## 3. Results

### 3.1. Normal Density Distribution

**Figure 5:**
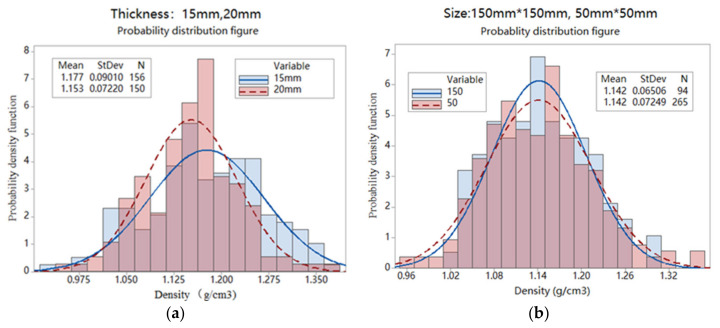
Density histograms for panels with (**a**) different panel thickness and (**b**) evaluation specimen size.

The distribution of density broken down by panel thickness (15 or 20 mm) and specimen size (50 × 50 or 150 × 150-mm^2^) are shown in Figure 5. Figure 5a,b also indicate the same average density but a greater spread of density values with the higher standard deviation (SD) in the thinner panel or density evaluated with smaller specimen size. The Anderson–Darling test was used to verify that all four data distributions were normal (or Gaussian).

The normal distributions stem from the Poisson distributions of local strip overlaps or densities formed at any given point in the plane of panel. Poisson principles were first discovered in wood composite mats [23,24] and may exist in BFCs, even though the bamboo strip mats are manually placed rather than machine-formed. The second reason for the normal distribution is that the measurements of density are based on finite areas which integrate an infinite number of points within those areas. Therefore, there is a natural averaging effect, making the distribution more bell-shaped than skewed.

With the normal distribution, the density data can then be adequately characterized by averages and standard deviation (SD). While the density average is usually governed by the product’s need for strength performance, SD is a function of strip geometry, panel thickness and the formation process. The smaller the SD value, the more uniform the BFC. In practice, SD or COV (normalized SD) can be used as an indicator for mat uniformity.

A reference normal distribution curve of density and acceptable variation can be pre-established for BFC products based on inputted control parameters for each product, such as bamboo species, panel thickness, element size and panel compaction ratio (panel density/bamboo density). As a means of quality control, weekly sampling of density specimens, ideally larger-sized specimens (e.g., 150 × 150-mm^2^), can be used to confirm whether panels conform to the reference average density and its standard deviation. From this ongoing monitoring of product processing, parameters can be further calibrated and optimized and eventually switched to an online, non-destructive density scanning method.

### 3.2. Comparing BFC with Wood Composites

Table 2 gives values from the literature of mean panel density and its variation (coefficient of variation, COV) for different machine-formed wood composites, including particle board, medium density fiberboard (MDF), waferboard and oriented strand board (OSB) [32]. The mean density and COV for the BFC made in this work are based on the 15-mm-thick panel and the 50 × 50-mm^2^ specimen size to most closely match the parameters used for the wood composite panels.

The first thing to note is the much higher density of BFC (1.15 g/cm^3^) compared with hot-pressed wood composites This is mainly due to a combination of the higher material density of Moso bamboo (0.67 g/cm^3^) compared with low-density woods commonly used in particle board and OSB (e.g., aspen at 0.43 g/cm^3^). Note, also, that both the BFC and wood composite densities were based on an air-dried rather than oven-dried weight basis. Generally speaking, high compaction is needed to create close contact between constituents for bonding, especially for composites made with discrete elements (i.e., fibers, particles, strands or strips) [20]. A preliminary analysis showed that the compaction ratios (density of composites/density of wood or bamboo) for BFC (1.72) are high compared with wood-based products: MDF (1.62), particle board (1.52) and OSB (1.56).

Table 2 shows that the small-element composites (MDF and PB) have the lowest COV (1–3%), followed by waferboard (4.7%) and OSB (6.6%). Waferboard differs from OSB in that it is made with smaller, shorter elements than OSB, which are partially directionally oriented and made with strands up to 100 mm in length. Smaller, more even-sized and more numerous elements in the mat make it easier to control the horizontal density distribution by machine mat forming. Note that BFC is made with much larger and thicker elements than OSB, yet the COV (6.7%) was almost the same, suggesting that the manual mat formation process can give a high degree of control over horizontal density variation in BFC panels.

### 3.3. Effects of Panel Thickness and Specimen Size

Average density, maximum and minimum values and variability (SD and COV values) for the 50 × 50-mm^2^ specimens from each panel are given in Table 3.

**Figure 6:**
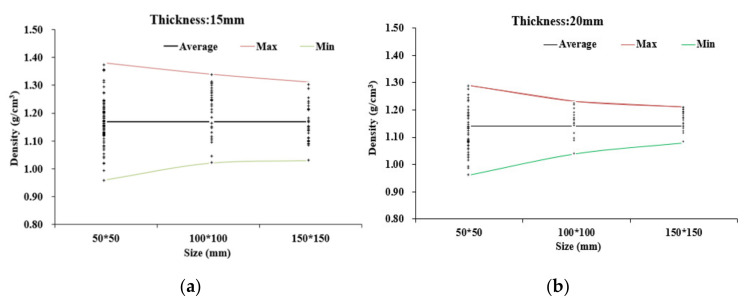
Spread of point densities by specimen size for (**a**) 15- and (**b**) 20-mm panels.

Figure 6 shows the spread of density values for each specimen size and thickness. The increase in spread is significant as specimen size decreased from 100 × 100 to 50 × 50-mm^2^. SD of density decreased from 0.078 g/cm^3^ for 50 × 50-mm specimens to 0.060 and 0.044 g/cm^3^ for 100 × 100 and 150 × 150-mm^2^ specimens, respectively. The narrowest range of density values was in the 20-mm-thick panel using the 150 × 150-mm^2^ specimen size, ranging from 1.08 to 1.21 g/cm^3^ (Figure 6b). As the specimen size decreased, the number of specimens sampled increased greatly from 36 per panel for 150 × 150-mm^2^ to 96 per panel for 50 × 50-mm^2^. Note that as the panel thickness increased, the spread of density readings reduced, particularly for the larger specimens—i.e., thin panels with fewer layers of flattened culm in the mat have greater planar density variation, as might be expected.

A simplified function adapted from Dai and Steiner [23], developed to predict density variance based on element size and panel thickness, provides a predictive model for SD, based on density specimen side length (a) and panel thickness (T), given below:

σ = f(a)/√T

**Figure 7:**
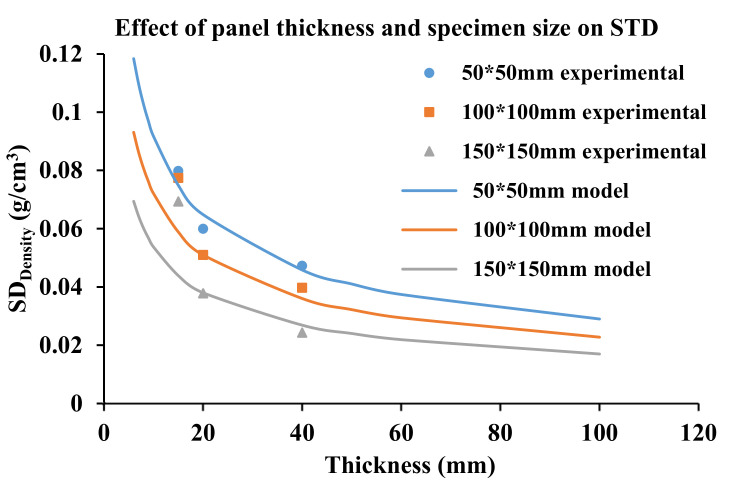
Density variability of BFC with panel thickness and specimen size.

Assuming uniformly random distribution, the model indicates that the standard deviation of density (SD) is a function of the specimen side length and inversely proportional to the square root of the panel thickness. The generated model values (solid lines) for 50 × 50, 100 × 100 and 150 × 150-mm^2^ specimens, with T ranging from 5 to 100 mm, are plotted in Figure 7. The average SD from the measured values for all nine groups (three specimen sizes and three panel thicknesses) is overlayed, demonstrating good agreement with the trends for density specimen size and panel thickness. Generally, both model and empirical data show that SD decreases with increasing panel thickness and specimen size, but there are discrepancies depending on the group, particularly thickness. Note that the model prediction for the 150 × 150-mm^2^ size fits experimental SD values for 20- and 40-mm-thick panels, but the experimental SD was much higher for 15-mm panels, despite their greater compaction, suggesting a greater heterogeneity in mat structure that is not picked up in the model for random effects of specimen size and thickness only. It suggests that 15 mm is not as efficient a panel thickness for BFC and that thicker panels with more layers may be preferable to overcome the random effects from smaller numbers of very large elements (i.e., bamboo culm strips). For 100 × 100-mm^2^ specimens, the experimental SD values fit the model in 15-mm panels but were lower in 20- and 40-mm panels. For 50-mm specimens, the experimental SD values were consistently above the model, likely because of the end/edge bias, but generally followed the curve trend.

A practical outcome from this work is that using variable specimen size to assess panel uniformity can help guide the quality control process during production, which can be incrementally adjusted to reduce the spread of density. From the data, the optimal specimen size is selected based on the density range of “reference” panels such as those carefully produced and destructively sampled here, and the density of a smaller number of selected specimens sampled from mass production can then be tested to verify that panel density falls within the expected range of reference density for its type, and any manufacturing flaws can be corrected if necessary. The variable specimen size method can be used to check the uniformity of density of the whole panel and evaluate the panel quality and properties of each batch. It provides an accurate and fast method for companies to adjust quality benchmarking during the manufacturing process, particularly if custom-fabricating products for a specific order. A sample of low-density specimens having relatively low SD indicates good product quality control. During manufacturing, larger specimens are easier to cut and measure, and this is less time-consuming and would, therefore, be preferred. For example, if the panel thickness is 20 mm and all values of measured density fall within the expected range of 1.08 and 1.21 g/cm^3^ for 150 × 150-mm^2^-size specimens, it may be considered that the overall panel density meets the reference product specifications.

### 3.4. Spatial Variation

To examine differences in density distribution from the panel edges to its centerlines, four sets of density data (edge-length, center-length, edge-width and center-width) for 50 × 50-mm^2^ density specimens were used to calculate SD, given in Table 4.

#### 3.4.1. Density Distribution along the Length of the Panel

**Figure 8:**
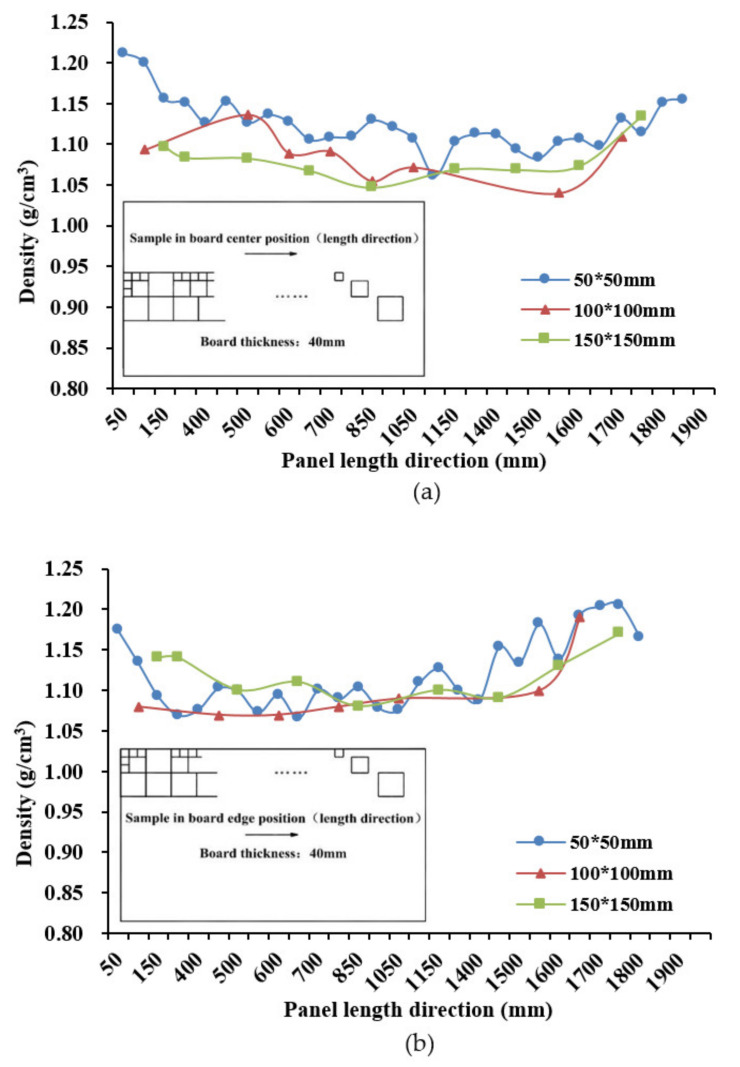
Density distribution along panel length for (**a**) centerline and (**b**) edge of panel.

Figure 8 shows the distribution in density along the length of the panel (parallel to bamboo fiber) of the 40-mm-thick panel along the edge (a) and center (b), with the different lines representing specimen size. Again, the small specimens picked up more of the localized variation in density along the panel, including peaks in density at either end of the panel, where the extra filler pieces were inserted. Density values were higher if sampled along the edge of the panel compared with the center due to the side-compacting effect from the mold edges during hot pressing. Note the directional upward or downward trend in density along the length of the panel, perhaps reflecting some bias in the culm orientation, especially along the edge.

#### 3.4.2. Density Distribution across the Width of the Panel

**Figure 9:**
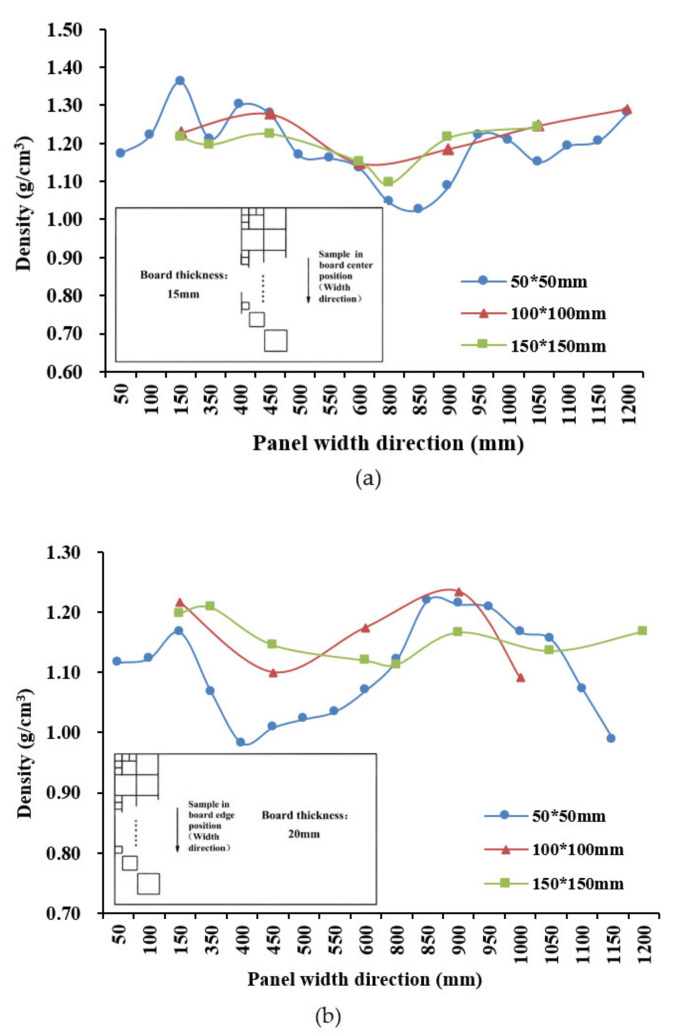
Density distribution across panel width for (**a**) centerline and (**b**) end of panel.

Figure 9 shows the change in density across the width (perpendicular to bamboo fiber direction) of the 15-mm panel (a) through the middle and (b) along the end. Again, the small specimens picked up more of the inherent variability in density across the panel, which was much greater at the end than across the middle. The selective insertion of extra strips into the ends of the mat led to greater variation in compaction and localized density at the ends. As might be expected, there was greater variability in local density across the bamboo strips. This is caused by strip overlaps and accompanying localized variability in the compaction ratio during pressing. The general increase in density towards the edges is most likely due to the lateral compression from the mold during hot pressing, as the mat expands horizontally but is constrained by the edges. There was little observed bias in density trends from one side of the panel to the other.

#### 3.4.3. Density Standard Deviation along and Cross the Panel

**Figure 10:**
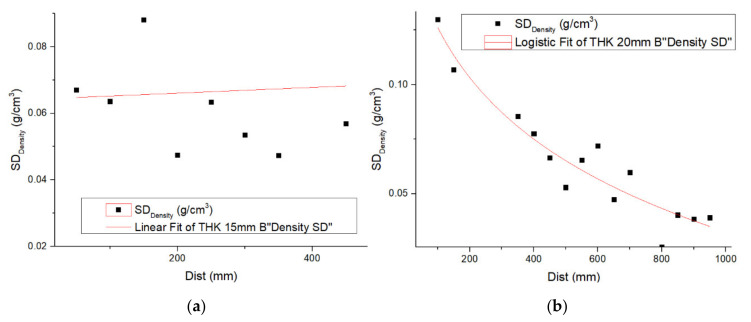
Change in SD of with distance from centerlines to edges for 20-mm panel. (**a**) With direction, (**b**) Length direction.

The effect of distance of the specimens from the edge of the panel along the center-width and center-length directions on SD is shown in Figure 10. Figure 10a shows no distinct trend in SD going from the long edge to the center, while Figure 10b shows significantly greater SD among the specimens taken from the ends of the panel compared with the middle transect across the width, as reflected in Table 4. In order to maintain consistency in group size regardless of the specimen size, the SD values in the plots were derived for four 50 × 50-mm^2^ specimens at each 50 × 50-mm^2^ interval going from the ends to the center in the case of length, and the same for width from the edges to the centerline of the panel.

The patterns are as expected since there is better control of density across the panel width through the mat lay-up technique due to the fact that the scrimmed culm strips are far less variable in thickness and density across their width than they are along their length. The greater variability in density at the ends is also contributed to by the possible insertion of filler pieces to ensure that the unrestrained ends are not lower in density after pressing. However, it is not known whether short strips were used in the panels sampled here. The ends were also unrestrained during hot pressing, another possible contributing factor. The change in thickness along the length of each flattened culm strip also contributes to greater unevenness in density along the length of panels, although there should be an even number of layers with culm strips running in both directions, as there is an even number of layers in each mat. Data from Yang [33] indicate a 5-mm decrease in culm wall thickness from the bottom to the top end of a 2-m-long Moso bamboo culm. The extra tissue in the lower part of the culm mostly occurs on the inner wall since the difference in outer diameter over the 2-m length is just 1–2 mm, and the wall thickening is greatest in the section at the base of the bamboo plant.

## 4. Conclusions

Full-sized bamboo fiber composite (BFC) mats were fabricated manually and hot pressed in a commercial factory at three thicknesses: 15, 20 and 40 mm. The horizontal density distributions (HDDs) were evaluated for three specimen sizes: 50 × 50, 100 × 100 and 150 × 150-mm^2^, and at different locations. The density values were normally distributed. The standard deviation (SD) of density decreased with increasing panel thickness due to improved consolidation of a greater number of layers of strips and also with increasing size of test specimens resulting from a greater averaging effect. Despite the manual forming process and large strip size, the BFC was shown to be relatively uniform in density compared with machine-formed wood composites. Notably, the SD for the BFC was almost the same as an OSB made from much smaller, machine-laid wood strands. The density at the long edges of the panels was higher due to the restraining effects of the mold edges during hot pressing. Specimens taken from the ends of panels were more variable in density due to thickness tapering of culm strips and the possible manual insertion of extra short strips into the ends to fill any noticeable gaps. For in-plant quality control protocols, 150 × 150-mm^2^ specimens may be used to sample and evaluate the density variation, and for a 20-mm-thick panel, the ideal product density range should be 1.08 and 1.21 g/cm^3^. Using the sampling technique here, similar optimum density ranges for the entire product range produced by a BFC facility may be established and used for quality control monitoring. BFC is best produced at a thickness greater than 15 mm to avoid excessive density variability. Further work will use discrete element modeling to simulate the HDD of BFC and analyze the full parametric effects of adjusting mat forming parameters such as number of strips and layers, panel thickness, strip thickness, thickness taper and direction and strip width.

## Figures and Tables

**Table 1:** Experiment design for density sampling.

Factor	Levels	N/Panel	Total
Specimen size	50 × 50-mm^2^	96	288
	100 × 100-mm^2^	38	114
	150 × 150-mm^2^	36	108

**Table 2:** Comparison of density variation of different commercial panel types.

Composite Type	MDF ^1^	PB Structural ^1^	PB Furniture ^1^	Wafer Board ^1^	OSB ^1^	BFC ^2^
Element	Fibers	Particles	Particles	Wafers	Strands	Strips
Thickness (mm)	16.3	12	16	11.1	9.5	15
Avg. density (g/cm^3^)	0.81	0.76	0.65	0.67	0.67	1.15
COV (%)	1.0	3.1	2.4	4.7	6.6	6.7

^1^ Kruse et al. (2000) [31]; ^2^ this work—15-mm-thick panel and 50 × 50-mm^2^ specimens. MDF— medium density fiberboard; OSB— oriented strand board; PB—particle board; COV—coefficient of variation.

**Table 3:** Average density and variability for each panel thickness (50 × 50-mm^2^ specimens).

Panel Thickness (mm)	15	20	40
Avg. Density (g/cm^3^)	1.15	1.13	1.10
Max	1.36	1.29	1.21
Min	0.96	0.98	1.04
SD	0.078	0.060	0.044
COV (%)	6.8	5.3	4.0

**Table 4:** Location effects on density and its variability for a 15-mm panel using 50 × 50-mm^2^ specimens.

15 mm Panel	Sampling Position			
Sampling Direction	E-L	C-L	E-W	C-W
Ave. density (g/cm^3^)	1.18	1.14	1.2	1.2
Max	1.33	1.27	1.36	1.36
Min	1.05	1.02	0.96	1.02
SD	0.054	0.062	0.114	0.084
COV (%)	0.045	0.055	0.094	0.069

## Data Availability

No new data were created or analyzed in this study. Data sharing is not applicable to this article.
